# Secondary metabolites and their bioactivities from *Paecilomyces gunnii* YMF1.00003

**DOI:** 10.3389/fmicb.2024.1347601

**Published:** 2024-02-19

**Authors:** Su-Su Li, Shuai-Ling Qu, Juan Xie, Dong Li, Pei-Ji Zhao

**Affiliations:** ^1^State key Laboratory for Conservation and Utilization of Bio-Resources in Yunnan, School of Life Sciences, Yunnan University, Kunming, Yunnan, China; ^2^The Maternal and Child Health Hospital of Qianxinan, Xingyi, Guizhou, China

**Keywords:** *Paecilomyces gunnii*, polyketides, cytotoxic activity, protein kinase Cα inhibitory activity, calculated electronic circular dichroism

## Abstract

Four new polyketides (**1**–**4**) and seven known compounds (**5**–**11**) including three polyketides and four sterols were isolated from the fermented extracts of *Paecilomyces gunnii* YMF1.00003. The new chemical structures were determined through the analysis of the nuclear magnetic resonance and high-resolution electrospray ionization mass spectrometry, and their configurations were subsequently confirmed by nuclear overhauser effect spectroscopy, the calculated electronic circular dichroism (ECD) spectra, and quantum chemical calculations of the NMR data (qcc NMR). Based on the results of pre-activity screening and compound structure target prediction, certain metabolites were assayed to evaluate their cytotoxic and protein kinase Cα inhibitory activities. Results indicated that 3β-hydroxy-7α-methoxy-5α,6α-epoxy-8(14),22*E*-dien-ergosta (**8**) exhibited potent cytotoxic activity, with half-maximal inhibitory concentration values of 3.00 ± 0.27 to 15.69 ± 0.61 μM against five tumor cells, respectively. The new compound gunniiol A (**1**) showed weak cytotoxic activity at a concentration of 40 μM. At a concentration of 20 μg/mL, compounds **1**, **6**, and **7** exhibited protein kinase Cα inhibition by 43.63, 40.93, and 57.66%, respectively. This study is the first to report steroids demonstrating good cytotoxicity and polyketides exhibiting inhibitory activity against protein kinase Cα from the extracts of *P. gunnii*.

## 1 Introduction

*Cordyceps*, a fungus known for parasitizing insects, fungi, and plants (Qu et al., 2022), gained historical recognition during the Qing Dynasty, with *Cordyceps sinensis* emerging as the most well-known species documented in Bencao Beiyao. An in-depth study of its pharmacological properties revealed immunomodulatory functions and a significant impact on antitumor effects, organ transplantation, and heart disease (Kuo et al., 1996). *Cordyceps gunnii*, a well-known fungus in the *Cordyceps* genus, exhibits diverse bioactivities.

**Figure 1:**
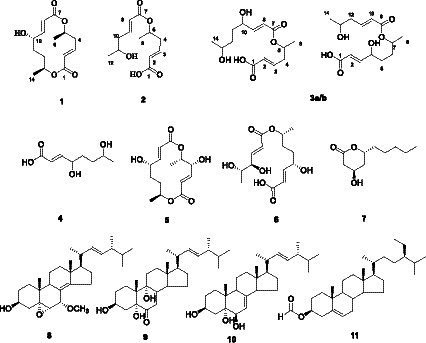
Structures derived from the *Paecilomyces gunnii* YMF1.00003.

*Paecilomyces gunnii*, an anamorph of *C. gunnii*, produces metabolites that exhibit various pharmacological activities. Three new metabolites, gunnilactams A-C, were isolated from the deep fermentation broth of *P. gunnii*, and gunnilactam A showed cytotoxic activity against C42B cells (human prostate cancer) with a half-maximal inhibitory concentration (IC_50_) of 5.4 μM (Zheng et al., 2017). Following the performance of activity-guided procedures and liquid chromatography–mass spectrometry (LC-MS), paecilomycones A–C were identified from *P. gunnii*, exhibiting strong tyrosinase inhibitory activity with IC_50_ values of 0.11, 0.17, and 0.14 μM, respectively (Lu et al., 2014). In addition, a new selenopolysaccharide (SeCPS-II), consisting of α-L-rhamnose, α-D-mannose, α-D-glucose, and β-D-galactose with a molecular weight of 4.12 × 10^3^ kDa, was obtained from *C. gunnii* and showed weak inhibition of SeCPS-II on SKOV-3 cells (Sun et al., 2018). The identified active compounds exhibited potential for various applications, and further in-depth research on them holds significant value in the fields of pharmacology and medicine. In a previous study, we examined the metabolome and the activity of extracts derived from the *P. gunnii* YMF1.00003 strain. Results showed significant variations in the activities of fermentation product extracts under various culture conditions. The extract obtained from the wheat bran medium (WGA) exhibited notable inhibitory activity against the tested cell lines, resembling the effects observed in the extract from the stroma and host complex of *C. gunnii* (Qu et al., 2022). In this study, we aimed to examine the chemical structures of four new polyketides and seven known compounds isolated from *P. gunnii* YMF1.00003 cultivated in two types of media (Figure 1). Based on the results of a previous study (Qu et al., 2022), extensive literature research, and the prediction of compound structure targets, we conducted assays on select compounds to assess their cytotoxic activity and protein kinase Cα inhibitory activity.

## 2 Materials and methods

### 2.1 General experimental procedures

Electrospray ionization mass spectra (ESI-MS) and high-resolution electrospray ionization mass spectra (HR-ESI-MS) were acquired using a high-resolution Q Exactive Focus Mass Spectrometer (Thermo Fisher Scientific, Bremen, Germany). Optical rotation measurements were conducted using a Jasco DIP-370 digital polarimeter (JASCO, Tokyo, Japan). Ultraviolet (UV) spectra were recorded using a Shimadzu UV-2401PC spectrophotometer. Nuclear magnetic resonance (NMR) spectra were recorded using an Avance III-600 spectrometer (Bruker BioSpin, Rheinstetten, Germany) using tetramethylsilane as an internal standard. Column chromatography was performed using silica gel (200–300 mesh), GF254 (Qingdao Marine Chemical Inc., Qingdao, China), and Sephadex LH-20 (Amersham Pharmacia, United Kingdom). Semipreparative high-performance liquid chromatography (HPLC) was performed using an LC3000 system (Beijing Chuangxintongheng Science and Technology Co., Ltd., Beijing, China). Precoated silica gel GF254 plates (Qingdao Marine Chemical Factory, Qingdao, China) were used for thin-layer chromatography (TLC) analysis.

### 2.2 Microbial strain, media, and cultivation

The *P. gunnii* YMF1.00003 strain was officially deposited at the State Key Laboratory for the Conservation and Utilization of Bio-Resources, Yunnan University, Kunming, China. *Paecilomyces gunnii* YMF1.00003 was fermented in two different media. During the pre-experiment, the solid and liquid forms of four different media (PDB, modified Sabouraud medium, Rice, and Oats) were used to determine the most suitable medium for the production of YMF1.00003 metabolites. Based on the amount of extracts and results of TLC analysis, the modified Sabouraud medium demonstrated a substantial amount of YMF1.00003 extracts, with a more diverse range of compounds. Consequently, the modified Sabouraud agar medium was selected for subsequent fermentation. The strain was cultured in 9-cm dishes with modified Sand’s solid medium (comprising 5.0 g of tryptone, 1.0 g of yeast extract, 60.0 g of glucose, 0.25 g of K_2_HPO_4_, 0.25 g of MgSO_4_, 0.25 g of KCl, 15.0 g of agar, and 1 L of water) at 28°C for 21 days and 30 L. The cultures were extracted using organic reagents (ethyl acetate*/*methanol*/*glacial acetic acid = 80:15:5, *v/v/v*; Pu et al., 2021; Liu et al., 2022) to obtain 23.9 g of crude extract and subsequently designated as the MSA fraction. In addition, the strain was cultured on a modified wheat bran medium (comprising 30.0 g of wheat bran, 20.0 g of glucose, 1.5 g of KH_2_PO_4_, 1.5 g of MgSO_4_, 0.5 g of MnSO_4_∙H_2_O, 0.5 g of ZnSO_4_∙7H_2_O, 0.5 g of CuSO_4_∙5H_2_O, 8.0 g of (NH_4_)_2_SO_4_, 15 g of agar, and 1 L of water) at 28°C for 50 days. Subsequently, 30 g of crude extract was obtained and designated as the WB fraction.

### 2.3 Isolation and purification

After mixing with silica 60 RP-C18, the MSA fraction (23.9 g) was placed on an RP-C18 column (50 g) and eluted with H_2_O/MeOH (100:0 → 0:100, *v*/*v*) to obtain 12 fractions (Fr.1–Fr.12). Fr.9 (1.617 g) was separated with a Sephadex LH-20 column (chloroform–methanol, 1:1) to obtain nine fractions (Fr.9.1-9). Fr.9.3 (1.254 g) was separated using a silica gel column (200–300 mesh) and eluted with petroleum ether/acetone (100:1 → 6:4, *v*/*v*) to obtain five fractions (Fr.9.3.1-5). Fr.9.3.2 (135 mg) was loaded on a silica gel column eluting with chloroform/acetone (100:1 → 6:4, *v*/*v*) and then purified by Sephadex LH-20 (acetone) to obtain compound **8** (3 mg). Fr.9.3.4 (180 mg) was subjected to Sephadex LH-20 chromatography (chloroform–methanol, 1:1) to obtain four fractions (Fr.9.3.4.1-4). Fr.9.3.4.3 (132 mg) underwent separation on a silica gel column eluted with chloroform/acetone (100:1 → 8:2, *v*/*v*) and was subsequently purified by a Sephadex LH-20 (acetone) to obtain compound **9** (2 mg). Fr.9.3.5 (113 mg) was loaded on a Sephadex LH-20 (acetone) and purified on a silica gel column via elution with chloroform/methanol (100:1 → 8:2, *v*/*v*) to obtain compound **10** (8 mg). Fr.5 (259 mg) was subjected to Sephadex LH-20 (chloroform–methanol, 1:1) chromatography to obtain five fractions (Fr.5.1-5). Fr.5.4 (54 mg) was further separated using a silica gel column via elution with chloroform/acetone (100:1 → 6:4, *v*/*v*) and purified by Sephadex LH-20 chromatography (acetone) to obtain compound **2** (7 mg). Fr.5.5 (80 mg) was subjected to silica gel column chromatography and eluted with chloroform/acetone (100:0 → 8:2, *v*/*v*) and purified by Sephadex LH-20 chromatography (methanol) to obtain compound **7** (5 mg). Fr.4 (1.6 g) was loaded on a Sephadex LH-20 column (chloroform–methanol, 1:1) to obtain seven fractions (Fr.4.1-7). Fr.4.5 (637 mg) was further separated using Sephadex LH-20 (methanol) to obtain four fractions (Fr.4.5.1-4). Fr.4.5.2 (439 mg) was subjected to semi-preparative HPLC with gradient elution of MeOH–H_2_O (30:70 → 35:65 and 40:60 → 100:0) for 50 min to obtain five fractions (Fr.4.5.2.1-5). Fr.4.5.2.3 (120 mg) was separated using a Sephadex LH-20 column (methanol) to obtain three fractions (Fr.4.5.2.3.1-3). Fr.4.5.2.3.3 (108 mg) was further separated using a silica gel column via elution with chloroform/acetone (100:1 → 0:100, *v*/*v*) to obtain eight fractions (Fr.4.5.2.3.3.1-8). Fr.4.5.2.3.3.6 (51 mg) was separated using a Sephadex LH-20 column (methanol) to obtain compound **6** (28 mg). Fr.4.5.2.5 (64 mg) was separated using a Sephadex LH-20 column (methanol) to obtain two fractions (Fr.4.5.2.5.1-2). Fr.4.5.2.5.2 (59 mg) was further separated using a column of silica gel via elution with chloroform/acetone (100:1 → 0:100, *v*/*v*) and purified by Sephadex LH-20 (methanol) to obtain compound **3a/b** (5 mg). Fr.3 (2.135 g) was separated using a Sephadex LH-20 column (chloroform–methanol, 1:1) to obtain five fractions (Fr.3.1-5). Fr.3-5 (1.232 g) was separated using a Sephadex LH-20 column (chloroform–methanol, 1:1) to obtain four fractions (Fr.3.5.1-4). Fr.3.5.4 (1.081 g) was subjected to semi-preparative RP-C18 HPLC with a gradient elution of MeOH:H_2_O (10:90 → 45:55 and 60:40 → 100:0) for 40 min to obtain three fractions (Fr.3.5.4.1-3). Fr.3.5.4.3 (34 mg) was further separated using a silica gel column via elution with chloroform/acetone (50:1 → 0:100, *v*/*v*) to obtain four fractions (Fr.3.5.4.3.1-4). Fr.3.5.4.3.2 (8 mg) was separated using a Sephadex LH-20 column (methanol) to obtain compound **4** (5 mg).

The WB fraction (30.0 g) was placed on an RP-C18 column (60 g) and eluted with H_2_O/MeOH mixtures (100:0 → 0:100, *v*/*v*) to obtain 15 fractions (Fr.1-15). Fr.15 (878 mg) was separated using a silica gel column via elution with petroleum ether/ethyl acetate (300:1 → 6:4, *v*/*v*) to obtain five fractions (Fr.15.1-5). Fr.15.2 (153 mg) was separated using a silica gel column via elution with petroleum ether/ethyl acetate (100:1 → 8:2, *v*/*v*) and then purified with a silica gel column via elution with petroleum ether/ethyl acetate (200:1 → 8:2, *v*/*v*) to obtain compound **11** (1 mg). Fr.15.4 (123 mg) was separated using a silica gel column via elution with chloroform/acetone (10:1 → 6:4, *v*/*v*) and then purified by Sephadex LH-20 (acetone) to obtain compound **8** (2 mg). Fr.6 (385 mg) was loaded on a Sephadex LH-20 column (methanol) to obtain six fractions (Fr.6.1-6). Fr.6.3 (108 mg) was subjected to a silica gel column (100:1 → 7:3, *v*/*v*) and purified by Sephadex LH-20 (methanol) to obtain compound **1** (6 mg). Fr.4 (1.15 g) was subjected to Sephadex LH-20 column (chloroform–methanol, 1:1) to obtain five fractions (Fr.4.1-5). Fr.4.1 (715 mg) was loaded on a silica gel column via elution with chloroform/acetone (100:1 → 8:2, *v*/*v*) to obtain four fractions (Fr.4.1-4). Fr.4.1.3 (83 mg) was subjected to a Sephadex LH-20 column (methanol) and purified by a silica gel column via elution with chloroform/acetone (100:1 → 8:2, *v*/*v*) to obtain compound **5** (15 mg).

### 2.4 Spectroscopic data

Gunniiol A (**1**), colorless solid; [α]^23^_D_ = 75.5 (*c* = 0.10, MeOH); UV (MeOH) λ_max_ (log ε) nm: 204 (4.46); ^1^H-NMR (CDCl_3_, 600 MHz) and ^13^C-NMR (CDCl_3_, 150 MHz), see Table 1; ESI-MS *m/z*: 291 [M + Na]^+^; HR-ESI-MS *m/z*: 291.1190 ([M + Na]^+^, calcd. 291.1203).

**Table 1:** The NMR data of gunniiols A (**1**) and B (**2**).

Position	Gunniiol A (**1**)			Gunniiol B (**2**)		
	^1^H	^13^C	HMBC	^1^H	^13^C	HMBC
1	-	165.5, s	-	-	170.6, s	-
2	5.79 (1H, d, 15.7)	126.3, d	C-3, C-4, C-1	5.89 (1H, d, 15.5)	123.8, d	C-1, C-4
3	6.71 (1H, ddd, 5.2, 10.7, 15.7)	143.4, d	C-1, C-4	6.97 (1H, m)	146.2, d	C-1, C-2, C-4, C-5
4	2.55 (1H, dt, 12.9, 3.8)	40.4, t	C-2, C-3, C-5	2.52 (2H, brs)	38.5, t	C-2, C-3, C-6, C-5
	2.29 (1H, dt, 10.7, 12.9)		C-2, C-3, C-5,C-6			
5	5.23 (1H, m)	68.5, d	-	5.11 (1H, m)	69.0, d	-
6	1.36 (3H, d, 6.3)	20.6, d	C-4, C-5	1.29 (3H, d, 6.3)	19.9, q	C-4, C-5
7	-	165.7, s	-	-	165.7, s	-
8	5.90 (1H, dd, 1.7, 15.7)	121.2, d	C-7, C-9, C-10	5.89 (1H, d, 15.5)	123.6, d	C-7, C-10
9	6.82 (1H, dd, 4.6, 15.9)	150.5, d	C-7, C-8, C-10	6.94 (1H, m)	145.5, d	C-7, C-10, C-11
10	4.60 (1H, brs)	70.3, d	-	2.36 (2H, t, 6.9)	41.8, t	C-8, C-9, C-11, C-12
11	1.96 (1H, m)	29.2, t	C-9, C-10, C-12	3.99 (1H, m)	66.8, d	-
	1.77 (1H, m)		C-9, C-12, C-13			
12	1.69 (1H, m)	26.3, t	C-10, C-11, C-14	1.23 (3H, d, 6.2)	23.2, q	C-10, C-11
	1.52 (1H, m)		-			
13	5.18 (1H, m)	69.2, d	C-11, C-14	**-**	**-**	**-**
14	1.19 (3H, d, 6.7)	17.7, d	C-12, C-13	**-**	**-**	**-**

Gunniiol B (**2**), colorless oil; [α]^23^_D_ = 22.5 (*c* = 0.10, MeOH); UV (MeOH) λ_max_ (log ε) nm: 197 (4.57); ^1^H-NMR (CDCl_3_, 600 MHz) and ^13^C-NMR (CDCl_3_, 150 MHz), see Table 1; ESI-MS *m/z*: 265 [M + Na]^+^; HR-ESI-MS *m/z*: 265.1040 ([M + Na]^+^, calcd. 265.1046).

Gunniiol C (**3a/b**), colorless oil; ^1^H-NMR (CD_3_OD, 600 MHz) and ^13^C-NMR (CD_3_OD, 150 MHz), see Table 2; ESI-MS *m/z*: 287 [M + H]^+^, 309 [M + Na]^+^; HR-ESI-MS *m/z*: 309.1300 ([M + Na]^+^, calcd. 309.1309).

**Table 2:** The NMR data of gunniiol C (**3a/b**).

Position		Gunniiol C (**a/b**)				
	^1^H	^13^C	HMBC	^1^H	^13^C	HMBC
1	-	167.8, s	-	-	167.8, s	-
2	5.89 (1H, d, 15.7)	124.5, d	C-1, C-4	6.00 (1H, d, 15.7)	120.8, d	C-1, C-4
3	6.83 (1H, m)	144.2, d	C-4, C-5	6.95 (1H, dd, 4.9, 15.7)	152.7, d	C-1, C-4
4	2.50 (2H, brt, 6.5)	39.3, t	C-2, C-3, C-5,C-6	4.23 (1H, m)	71.7, d	-
5	5.08 (1H, tq, 6.2, 6.3)	70.9, d	-	1.54 (2H, m)	33.5, t	C-4, C-6, C-7
6	1.28 (3H, d, 6.3)	20.3, q	C-4, C-5	1.63 (1H, m)	32.9, t	C-5, C-7
				1.71 (1H, m)		C-5, C-7
7	-	167.8, s	-	4.96 (1H, tq, 6.2, 6.1)	72.2, d	-
8	5.98 (1H, d, 15.7)	120.8, d	C-7, C-10	1.24 (3H, d, 6.2)	20.0, q	C-6, C-7
9	6.95 (1H, dd, 4.9, 15.7)	152.7, d	C-7, C-10	-	167.6, s	-
10	4.23 (1H, dt, 5.8, 4.9)	71.5, d	-	5.89 (1H, d, 15.6)	124.5, d	C-9, C-12
11	1.54 (2H, m, overlap)	33.9, t	C-10, C-12, C-13	7.00 (1H, dd, 7.4, 15.6)	147.3, d	C-9, C-12′
12	1.54 (2H, m, overlap)	35.9, t	C-10, C-11, C-13	2.34 (2H, m)	42.6, t	C-10, C-11, C-13, C-14
13	3.73 (1H, q, 6.4)	68.3, d	-	3.89 (1H, tq, 6.2, 6.2)	67.5, d	-
14	1.15 (3H, d, 6.2)	23.5, q	C-12, C-13	1.18 (3H, d, 6.2)	23.5, q	C-12, C-13

(*E*)-4,7-dihydroxyoct-2-enoic acid (**4**), colorless solid; [α]^23^_D_ = 19.4 (*c* = 0.10, MeOH); UV (MeOH) λ_max_ (log ε) nm: 198 (4.33); ^1^H-NMR (CD_3_OD, 600 MHz) and ^13^C-NMR (CD_3_OD, 150 MHz), see Table 3; ESI-MS *m/z*: 173 [M − H]^−^; HR-ESI-MS *m/z*: 173.0808 ([M − H]^−^, calcd. 173.0808).

**Table 3:** The NMR data of compound **4.**

Position	^1^H	^13^C	HMBC	COSY
1	-	170.2, s	-	-
2	5.98 (1H, d, 15.6)	121.3, d	C-1, C-4	H-3
3	6.89 (1H, dd, 15.6, 5.2)	152.3, d	C-1, C-4	H-2, H-4
4	4.23 (1H, m)	71.7, d	-	H-3, H-5
5	1.54 (1H, m)	34.0, t	C-7	H-4, H-6
	1.70 (1H, m)		C-6, C-7	
6	1.54 (1H, m)	35.9, t	C-7	H-5, H-7
	1.47 (1H, m)		C-5, C-7, C-8	
7	3.73 (1H, dq, 6.2, 6.2)	68.6, d	-	H-6, H-8
8	1.16 (3H, d, 6.2)	23.5, q	C-6, C-7	H-7

Clonostachydiol (**5**), colorless solid; ESI-MS *m/z*: 307 [M + Na]^+^; ^1^H-NMR (600 MHz, CD_3_OD) *δ*: 6.83 (1H, dd, *J =* 15.6, 4.0 Hz), 6.76 (1H, d, *J =* 15.6, 3.2 Hz), 6.14 (1H, t, *J =* 15.6, 1.6 Hz), 5.85 (1H, dd, *J =* 15.6, 1.6 Hz), 5.25 (1H, dq, *J =* 6.4, 2.0 Hz), 5.14 (1H, m), 4.57 (1H, m), 4.41 (1H, m), 1.95 (1H, m), 1.75 (1H, m), 1.64 (1H, m), 1.51 (1H, m), 1.41 (3H, d, *J =* 6.3 Hz), and 1.20 (3H, d, *J =* 6.6 Hz); ^13^C-NMR (150 MHz, CD_3_OD) *δ*: 167.3 (s), 167.0 (s), 153.5 (d), 148.4 (d), 125.1 (d), 121.7 (d), 77.2 (d), 73.0 (d), 71.2 (d), 70.6 (d), 29.7 (t), 27.1 (t), 17.9 (q), and 17.8 (q).

7*R*-[[4R,5S-dihydroxy-1-oxo-2*E*-hexen-1-yl]oxy]-4*S*-hydroxy-2*E*-octenoic acid (**6**), colorless oil; ESI-MS *m/z*: 325 [M + Na]^+^; ^1^H-NMR (600 MHz, CD_3_OD) *δ*: 5.99 (1H, d, *J* = 15.9 Hz, H-2), 6.91 (1H, dd, *J* = 4.8, 15.9 Hz, H-3), 4.25 (1H, m, H-4), 1.56 (1H, m, H-5a), 1.67 (2H, m, H-5b/6a), 1.75 (1H, m, H-6b), 5.00 (1H, m, H-7), 1.26 (3H, d, *J* = 6.4 Hz, H-8), 6.08 (1H, d, *J* = 15.9 Hz, H-2′), 7.07 (1H, dd, *J* = 4.8, 15.9 Hz, H-3′), 4.07 (1H, t, *J* = 4.8 Hz, H-4′), 3.72 (1H, t, *J* = 6.4 Hz, H-5′), and 1.18 (3H, d, *J* = 6.5 Hz, H-5b/H-6′); ^13^C-NMR (150 MHz, CD_3_OD) *δ*: 170.3 (s, C-1), 121.6 (d, C-2), 152.1 (d, C-3), 71.4 (d, C-4), 32.9 (t, C-5), 33.2 (t, C-6), 72.5 (d, C-7), 20.4 (q, C-8), 167.9 (s, C-1′), 122.7 (d, C-2′), 149.5 (d, C-3′), 76.2 (d, C-4′), 71.3 (d, C-5′), and 19.1 (q, C-6′).

(3*R*,5*R*)-3-hydroxy-5-decanolide (**7**), colorless oil; ESI-MS *m/z*: 209 [M + Na]^+^; ^1^H-NMR (600 MHz, CDCl_3_) *δ*: 4.72 (1H, dm, *J* = 11.2 Hz, H-5), 4.44–4.48 (1H, m, H-3), 2.78 (1H, dd, *J* = 17.7, 5.2 Hz, H-2a), 2.66 (1H, ddd, *J* = 17.7, 3.8, 1.6 Hz, H-2b), 1.99 (1H, dm, *J* = 14.7 Hz, H-4a), 1.78 (1H, ddd, *J* = 14.7, 11.2, 3.8 Hz, H-4b), 1.72–1.78 (2H, m, H-6), 1.30–1.34 (6H, m, H-7/8/9), and 0.93 (3H, t, *J* = 6.5 Hz, H-10); ^13^C-NMR (150 MHz, CDCl_3_) *δ*: 170.7 (s, C-1), 38.8 (t, C-2), 62.9 (d, C-3), 36.2 (t, C-4), 76.1 (d, C-5), 34.8 (t, C-6), 24.8 (t, C-7), 31.8 (t, C-8), 22.8 (t, C-9), and 14.2 (q, C-10).

3β-hydroxy-7α-methoxy-5α,6α-epoxy-8(14),22*E*-dien-ergosta (**8**), colorless oil; ESI-MS *m/z*: 443 [M + H]^+^, 465 [M + Na]^+^; ^1^H-NMR (600 MHz, CDCl_3_,) *δ*: 3.96 (1H, tt, *J* = 11.3, 4.7 Hz, H-3), 3.24 (1H, d, *J* = 2.7 Hz, H-6), 4.20 (1H, d, *J* = 2.1 Hz, H-7), 1.05 (3H, d, *J* = 6.7 Hz, H-21), 5.28 (2H, m, H-22/23), 0.89 (6H, s, H-18/19), 0.86 (3H, d, *J* = 6.5 Hz, H-26), 0.87 (3H, d, *J* = 6.8 Hz, H-27), 0.96 (3H, d, *J* = 6.7 Hz, H-28), and 3.45 (3H, s, 7-OCH_3_); ^13^C-NMR (150 MHz, CDCl_3_) *δ*: 153.6 (q, C-14), 135.6 (d, C-22), 132.5 (d, C-23), 122.8 (q, C-8), 72.9 (d, C-7), 69.0 (d, C-3), 65.5 (q, C-9), 40.2 (t, C-4), 39.6 (d, C-20), 36.8 (t, C-12), 36.3 (q, C-10), 33.3 (d, C-25), 32.5 (t, C-1), 31.5 (t, C-2), 27.5 (t, C-16), 25.2 (t, C-15), 21.5 (q, C-21), 20.2 (q, C-27), 19.9 (q, C-26), 19.5 (t, C-11), 18.5 (q, C-18), 17.9 (q, C-28), and 16.8 (q, C-19).

3β,5α,9α-trihydroxy-ergosta-7,22-dien-6-one (**9**), white powder; ESI-MS *m/z*: 467 [M + Na]^+^; ^1^H-NMR (600 MHz, CDCl_3_) *δ*: 4.09 (1H, m, H-3), 5.69 (1H, s, H-7), 1.06 (3H, s, H-18), 0.65 (3H, s, H-19), 1.05 (3H, d, *J* = 6.8 Hz, H-21), 5.20 (1H, dd, *J* = 15.4, 8.0 Hz, H-22), 5.23 (1H, dd, *J* = 15.4, 7.8 Hz, H-23), 0.85 (3H, d, *J* = 6.8 Hz, H-26), 0.87 (3H, d, *J* = 6.7 Hz, H-27), and 0.95 (3H, d, *J* = 6.8 Hz, H-28); ^13^C-NMR (150 MHz, CDCl_3_) *δ*: 25.5 (t, C-1), 26.4 (t, C-2), 67.4 (d, C-3), 33.8 (t, C-4),79.9 (s, C-5), 197.8 (d, C-6), 120.1 (d, C-7), 164.5 (s, C-8), 74.9 (d, C-9), 42.0 (s, C-10), 28.9 (t, C-11), 35.1 (t, C-12), 45.5 (s, C-13), 51.9 (d, C-14), 22.6 (t, C-15), 28.0 (t, C-16), 56.3 (d, C-17), 21.3 (q, C-18), 12.5 (q, C-19), 40.5 (d, C-20), 19.9 (q, C-21), 135.4 (d, C-22), 132.6 (d, C-23), 42.9 (d, C-24), 33.4 (d, C-25), 20.3 (q, C-26), 20.5 (q, C-27), and 17.8 (q, C-28).

3β,5α,6β-triol-7,22*E*-diene-ergosta (**10**), white powder; ESI-MS *m/z*: 453 [M + Na]^+^; ^1^H-NMR (600 MHz, C_5_D_5_N) *δ*: 4.85 (1H, m, H-3), 4.23 (1H, d, *J* = 10.2 Hz, H-6), 5.74 (1H, m, H-7), 0.65 (3H, s, H-18), 1.53 (3H, s, H-19), 1.05 (3H, d, *J* = 6.7 Hz, H-21), 5.14 (1H, dd, *J* = 15.3, 8.2 Hz, H-22), 5.21 (1H, dd, *J* = 15.3, 7.5 Hz, H-23), 0.84 (3H, d, *J* = 3.2 Hz, H-26), 0.85 (3H, d, *J* = 3.1 Hz, H-27), and 0.94 (3H, d, *J* = 6.8 Hz, H-28); ^13^C-NMR (150 MHz, C_5_D_5_N) *δ*: 32.7 (t, C-1), 33.8 (t, C-2), 67.6 (d, C-3), 42.0 (t, C-4), 76.1 (s, C-5), 74.3 (d, C-6), 120.5 (d, C-7), 141.6 (s, C-8), 43.7 (d, C-9), 38.1 (s, C-10), 22.4 (t, C-11), 39.9 (t, C-12), 43.8 (s, C-13), 55.3 (d, C-14), 23.5 (t, C-15), 28.5 (t, C-16), 56.1 (d, C-17), 12.5 (q, C-18), 18.8 (q, C-19), 40.9 (d, C-20), 21.2 (q, C-21), 136.2 (d, C-22), 132.1 (d, C-23), 43.1 (d, C-24), 33.3 (d, C-25), 19.9 (q, C-26), 20.2 (q, C-27), and 17.8 (q, C-28).

Stigmast-5-ene-3β-yl formate (**11**), colorless solid; ESI-MS *m/z*: 465 [M + Na]^+^; ^1^H-NMR (600 MHz, CDCl_3_) *δ*: 8.04 (1H, s, H-30), 4.73 (1H, m, H-3), 1.84 (1H, m, H-16), 1.57 (1H, m, H-1), 1.56 (1H, m, H-15), 1.46 (1H, m, H-11), 1.35 (1H, m, H-20), 1.32 (1H, m, H-22), 1.17 (1H, m, H-12), 1.10 (1H, m, H-17), 0.95 (1H, m, H-9), 0.93 (3H, d, *J =* 4.9 Hz, H-21), 0.84 (3H, d, *J =* 3.9 Hz, H-26), 0.83 (3H, d, *J =* 6.2 Hz, H-27), and 0.82 (3H, d, *J =* 5.0 Hz, H-29); ^13^C-NMR (150 MHz, CDCl_3_) *δ*: 36.9 (C-1), 27.8 (C-2), 74.0 (C-3), 38.1 (C-4), 139.3 (C-5), 123.0 (C-6), 31.9 (C-7), 31.8 (C-8), 50.0 (C-9), 36.6 (C-10), 21.0 (C-11), 39.7 (C-12), 42.3 (C-13), 56.7 (C-14), 24.3 (C-15), 28.2 (C-16), 56.0 (C-17), 19.3 (C-18), 11.9 (C-19), 36.1 (C-20), 18.8 (C-21), 33.9 (C-22), 26.0 (C-23), 45.8 (C-24), 29.1 (C-25), 19.8 (C-26), 12.0 (C-27), 23.0 (C-28), 19.0 (C-29), and 160.7 (C-30).

### 2.5 Cytotoxic activity

The cytotoxic activity of several compounds was assessed by conducting an assay using MTS, which is a new type of MTT analog. The method is often regarded as a “one-step” MTT assay as it allows the addition of reagents directly to cell cultures without the intermittent steps required in MTT assays. MTS holds an advantage over XTT owing to its increased solubility and non-toxic nature, enabling the reintroduction of cells to culture for further evaluation. The test concentration of each compound was 40 μM; for compounds with enhanced activity, a comprehensive screening of their activities at varying concentrations was conducted. Following the methods outlined in the literature (Su et al., 2013), five distinct cell lines (leukemia cell line HL-60, liver cancer cell line SMMC-7721, lung adenocarcinoma cell line A549, breast cancer cell line MDA-MB-231, and colon cancer cell line SW480) were selected for analysis. Cisplatin (40 μM) and paclitaxel (5 μM) were used as positive controls. All experiments were conducted in triplicate, and data were expressed as the mean ± standard deviation of three independent experiments.

### 2.6 Inhibitory activity against protein kinase Cα

According to the existing literature, certain types of polyketides showed antimicrobial bioactivity (Krohn et al., 2007). This finding suggests a limited number of studies exploring the activity of such compounds. To explore other activities of this type of polyketide, SwissTargetPrediction (STP)^1^ (accessed on 31 May 2022) was employed to identify potential targets associated with these metabolites (Gfeller et al., 2014). The results indicated that some metabolites may inhibit the activity of protein kinase Cα. Subsequently, compounds **1**, **2**, **3a/b**, **5, 6,** and **7** were evaluated to ascertain their protein kinase Cα inhibitory activity, using an enzyme-linked immunosorbent assay (ELISA) kit (Human Protein Kinase C, PKC ELISA Kit; Product number: kt80184, Wuhan Mosak Biotechnology Co., Ltd.). The experiment was conducted as follows: For sample addition, 50 μL of the standard sample was added to the wells in the microplate-coated plate, including five concentration points and 10 wells; the test samples included 10 μL of sample and 40 μL of diluent, with distilled water as the control. For incubation, the sample was incubated at 37°C for 30 min. For washing, each well was filled with washing solution, kept for 30 s, and subsequently discarded; the procedure was repeated five times. The enzyme was added (50 μL of enzyme-labeled reagent added to each well including the control), incubated, and washed (same as above). For color development, chromogenic agent A from the kit was initially added to each well, followed by the addition of 50 μL of chromogenic agent B from the same kit. The chromogenic agent was then incubated at 37°C in the dark for 15 min. For the termination stage, a termination solution (50 μL) was added to each well. For the determination stage, the absorbance (OD) of each well was measured at 450 nm within 15 min after adding the termination solution.

## 3 Results and discussion

### 3.1 Structural identification

**Figure 2:**
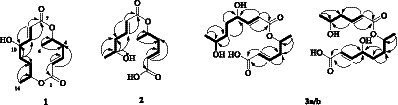
Selected HMBC (arrows) and ^1^H-^1^H COSY (bold bond) correlations of compounds **1–3**.

Compound **1** is a colorless solid with a molecular weight of 291.1190 [M + Na]^+^ based on the results of high-resolution mass spectrometry. The molecular formula is determined as C_14_H_20_O_5_. The planar structure of compound **1** was determined based on the key correlations of the two-dimensional nuclear magnetic resonance spectroscopy (2D NMR) data (Table 1): ^1^H−^1^H COSY spectra revealed the correlations between H-2/H-3/H-4/H-5/H-6, H-8/H-9/H-10/H-11/H-12/H-13/H-14, and −C-2–C-3–C-4–C-5–C-6− and −C-8–C-9–C-10–C-11–C-12–C-13–C-14− fragments (Figure 2). Additionally, long-range heteronuclear multiple bond correlations (HMBCs) revealed specific associations: H-2 (δ_H_ 5.79) was correlated with C-1 (δ_C_ 165.5), C-3 (δ_C_ 143.4), and C-4 (δ_C_ 40.4); H-3 (δ_H_ 6.71) was correlated with C-1 (δ_C_ 165.5) and C-4 (δ_C_ 40.4); H-6 (δ_H_ 1.36) was correlated with C-4 (δ_C_ 40.4) and C-5 (δ_C_ 68.5); H-9 (δ_H_ 6.82) was correlated with C-7 (δ_C_ 165.7), C-8 (δ_C_ 121.2), and C-10 (δ_C_ 70.3); H-13 (δ_H_ 5.18) was correlated with C-11 (δ_C_ 29.2) and C-14 (δ_C_ 17.7); and H-14 (δ_H_ 1.19) was correlated with C-12 (δ_C_ 26.3) and C-13 (δ_C_ 69.2; Figure 2). The flat structure of compound **1** mirrored that of colletallol (Kauloorkar and Kumar, 2016). After carefully analyzing the spectral data of compound **1** and colletallol, notable discrepancies were observed in the chemical shift of certain carbons, particularly C-14. In compound **1**, the chemical shift differences were δ_C_ 17.7 for C-14 (Table 1) and δ_C_ 19.5 for colletallol. In addition, the specific rotation value of compound **1** was [α]^23^_D_ = 75.5 (*c* = 0.10, MeOH), while the specific rotation of colletallol was [α]^23^_D_ = −107.5 (*c* = 0.40, CH_2_Cl_2_).

**Figure 3:**
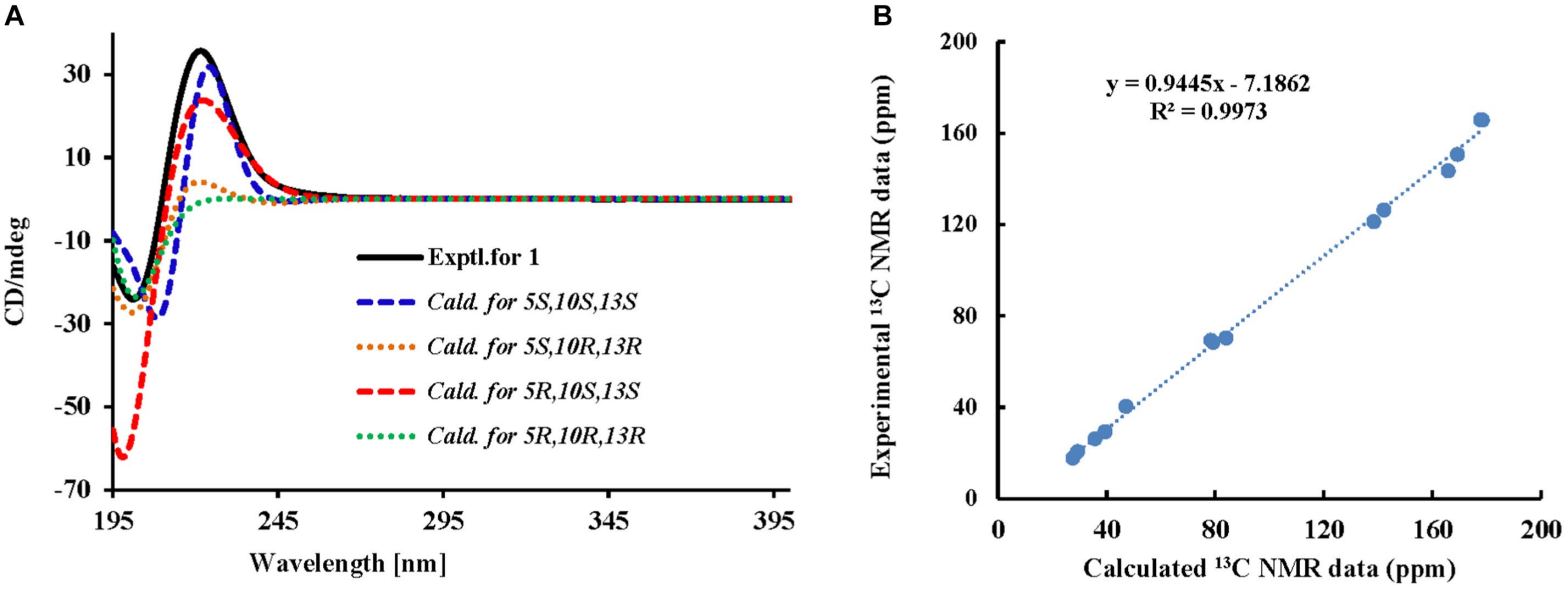
Experimental and calculated ECD curves and correlation plots of experimental and ^13^C NMR data of compound **1**. **(A)** Experimental and calculated ECD curves; **(B)** Correlation plots of experimental and calculated ^13^C NMR data.

Among them, H-14 exhibited an NOE effect with H-12β and H-10. The configuration of 6-CH_3_ cannot be determined by NMR data, so the calculated ECD spectra and qcc NMR of four stereoisomers, 5*S*,10*S*,13*S* (**1a**), 5*S*,10*R*,13*R* (**1b**), *5R,10S,13S* (**1c**), and 5*R*,10*R*,13*R* (**1d**), were performed. As given in Figure 3A, two cotton effects (CEs) with alternative signals were observed on the experimental spectrum. The 5*S*,10*S*,13*S* (**1a**) and *5R,10S,13S* (**1c**) theoretical spectra exhibited two CEs with alternative signs, which was in good agreement with the experimental ECD spectra. Moreover, the two epimers, 5*S*,10*S*,13*S* (**1a**) and *5R,10S,13S* (**1c**) were subjected to a strict conformational screening procedure, and the NMR chemical shifts were calculated at the mPW1PW91/6–31 + G(d,p)//M06-2X/def2-SVP level of theory with the PCM solvent model in methanol. The ^13^C NMR data with the correlation coefficient (R^2^) of 0.9973 of *5R,10S,13S* (**1c**) were consistent with its experimental values (Figure 3B). So the absolute configuration of compound **1** was assigned as *5R,10S,13S* (Figure 2) and was named gunniiol A.

Compound **2** is colorless oil, and its molecular formula C_12_H_18_O_5_ was determined by HR-ESI-MS with *m/z* 265.1040 ([M + Na]^+^, calcd for: 265.1046), with four unsaturations. In the ^13^C-NMR and DEPT analysis (Table 1), the carbon signals of compound **2** appeared in pairs and were characterized by fatty acid chains (δ_C_ 170.6, 165.7, 146.2, 145.5, 123.8, 123.6, 38.5, 41.8, 69.0, 66.8, 19.9, and 23.2), which was preliminarily judged to be a polyketone compound composed of one or two fatty acids.

The structural units of the two long chains, H-2/H-3/H-4/H-5/H-6 and H-8/H-9/H-10/H-11/H-12, were determined through the ^1^H-^1^H COSY experiment of compound **2,** revealing the structural motifs −C-2–C-3–C-4−C-5−C-6– and −C-8–C-9–C-10−C-11–C-12– (Figure 2). The HMBC data were used to determine the planar structure of compound **2**: H-2 (δ_H_ 5.89) was correlated with C-1 (δ_C_ 170.6) and C-4 (δ_C_ 38.5); H-3 (δ_H_ 6.97) was correlated with C-1 (δ_C_ 170.6), C-2 (δ_C_ 123.8), C-4 (δ_C_ 38.5), and C-5 (δ_C_ 69.0); H-6 (δ_H_ 1.29) was correlated with C-4 (δ_C_ 38.5) and C-5 (δ_C_ 69.0); H-9 (δ_H_ 6.94) was correlated with C-7 (δ_C_ 165.7), C-10 (δ_C_ 41.8), and C-11 (δ_C_ 66.8); H-10 (δ_H_ 2.36) was correlated with C-8 (δ_C_ 123.6), C-9 (δ_C_ 145.5), C-11 (δ_C_ 66.8), and C-12 (δ_C_ 23.2); and H-12 (δ_H_ 1.23) was correlated with C-10 (δ_C_ 41.8) and C-11 (δ_C_ 66.8). The chemical shift of H-5 exhibited a notable 5.11 ppm shift to the low field, suggesting the connection of the two polyketide chains through 5-OH and C-7 ester bonds. The structure of compound **2** was determined as shown in Figure 2 and named gunniiol B.

Compound **3a/b** is colorless oil. Its molecular formula was determined to be C_14_H_24_O_6_ (*m/z* 309.1300 [M + Na]^+^, calcd. 309.1309), with four unsaturations, by positive ion mode HR-ESI-MS. According to ^13^C-NMR and DEPT data (Table 2), the carbon signals of compound **3a/b** indicated paired appearances, akin to compound **2**, suggesting a polyketide. When the ^1^H- and ^13^C NMR spectra were analyzed, one fatty acid exhibited a similarity with compound **2**, identified as 5-hydroxyhex-2*E*-enoic acid (δ_C_ 167.8/167.6, 124.5, 144.2/147.3, 39.3/42.6, 70.9/67.5, and 20.3/23.5). Another fatty acid chain, 4,7-dihydroxyoct-2*E*-enoic acid (δ_C_ 167.8, 120.8, 152.7, 71.5, 33.9/33.5, 35.9/32.9, 68.3/72.2, and 23.5/20.0), was also identified in the analysis (Table 2).

In the ^1^H-^1^H COSY experiment, clear correlations were observed between the two structural units H-2/H-3/H-4/H-5/H-6 and H-8/H-9/H-10/H-11/H-12/H-13/H-14, the structural units of the two long chains −C-2–C-3–C-4−C-5−C-6− and −C-8–C-9–C-10−C-11−C-12–C-13−C-14− (Figure 2) or H-2/H-3/H-4/H-5/H-6/H-7/H-8 and H-10/H-11/H-12/H-13/H-14, and the structural units of the two long chains −C-2–C-3–C-4−C-5−C-6–C-7−C-8− and −C-10−C-11−C-12–C-13−C-14−. HMBC analysis confirmed the planar structure of compound **3a/b** (Figure 2). The two polyketide chains were linked in two ways: The chemical shift of H-5 to the low field reached δ_H_ 5.08 ppm, indicating that the two polyketide chains were connected by 5-OH and C-7 ester bonds. Alternatively, the chemical shift of H-7 to the low field reached δ_H_ 4.96 ppm, suggesting that the two polyketide chains were connected by 7-OH and C-9 ester bonds. The structures of compound **3a/b** were determined (Figure 2) and designated as gunniiol C.

Compound **4** is a colorless solid. Its molecular formula was determined to be C_8_H_14_O_4_ (*m/z* 173.0808 [M − H]^−^, calcd. 173.0808) with two degrees of unsaturation, by negative ion mode HR-ESI-MS. According to the ^13^C-NMR and DEPT (Table 3) results, the carbon signals of compound **4** were similar to those of compound **1** (Table 1). Careful analysis of the NMR data revealed that compound **4** was one of the two polyketone chains of compound **1** and was 4,7-dihydroxyoct-2*E*-enoic acid. The structure of compound **4** was elucidated through the analysis of HMBC: H-2 (δ_H_ 5.98) was associated with C-1 (δ_C_ 170.2) and C-4 (δ_C_ 71.7); H-3 (δ_H_ 6.89) was associated with C-4 (δ_C_ 71.7) and C-1 (δ_C_ 170.2); H-6 (δ_H_ 1.54 and 1.47) was associated with C-5 (δ_C_ 34.0) and C-7 (δ_C_ 68.6); and H-8 (δ_H_ 1.16) was associated with C-6 (δ_C_ 35.9) and C-7 (δ_C_ 68.6). The structure of compound **4** was determined as 4,7-dihydroxyoct-2*E*-enoic acid (Figure 1).

The other compounds were identified as clonostachydiol (**5**) (Ojima et al., 2018), 7*R*-[[4*R*,5*S*-dihydroxy-1-oxo-2*E*-hexen-1-yl]oxy]-4*S*-hydroxy-2*E*-octenoic acid (**6**) (Ojima et al., 2018), (3*R*,5*R*)-3-hydroxy-5-decanolide (**7**) (Wu et al., 2009), 3β-hydroxy-7α-methoxy-5α,6α-epoxy-8(14),22*E*-dien-ergosta (**8**) (Wu et al., 2015), 3β,5α,9α-trihydroxy-ergosta-7,22-dien-6-one (**9**) (Cai et al., 2013), 3β,5α,6β-triol-7,22*E*-diene-ergosta (**10**) (Zhao et al., 2010), and stigmast-5-ene-3β-yl formate (**11**) (Chumkaew et al., 2010). These identifications were made by comparing the obtained data with the information reported in the respective references.

### 3.2 Cytotoxic activity

**Figure 4:**
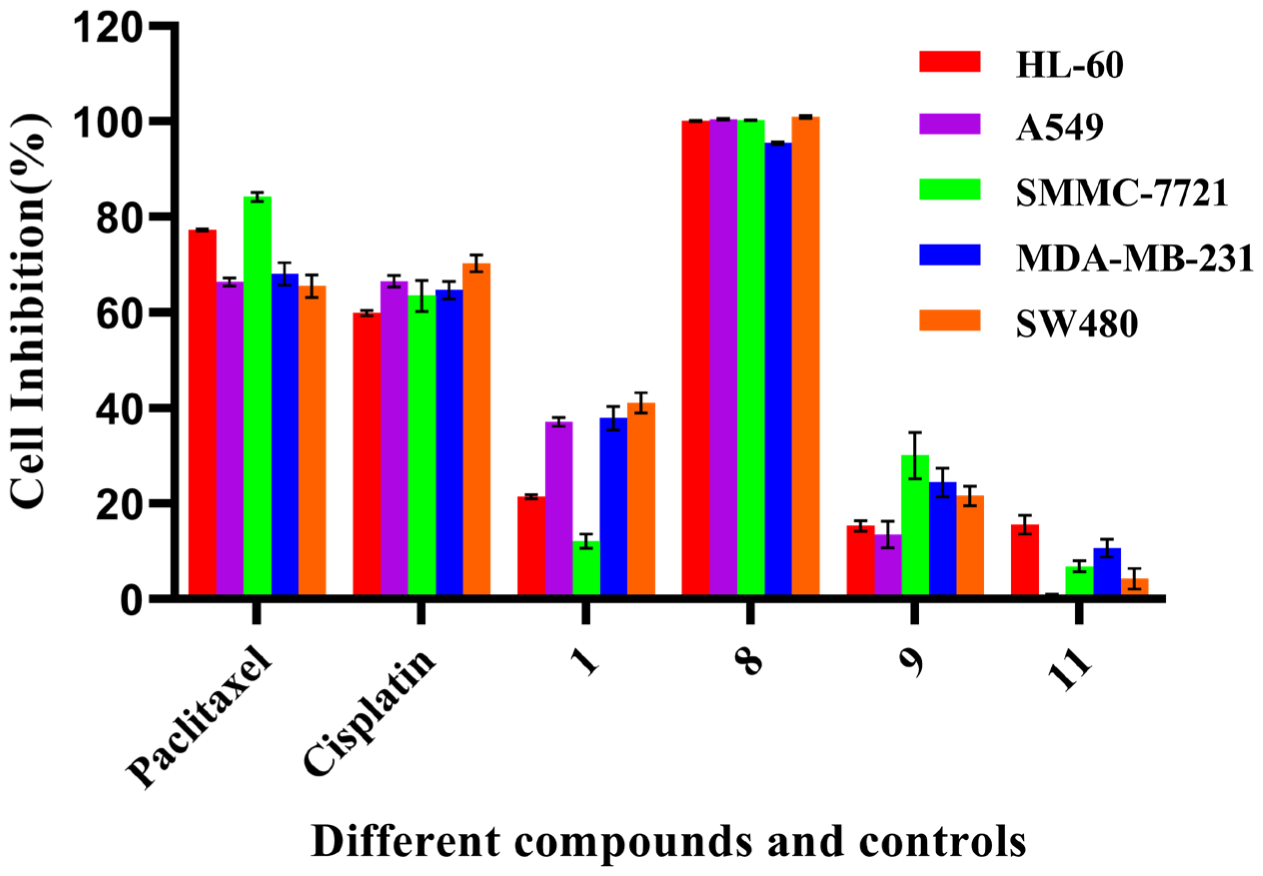
Cytotoxic activity of some compounds. The test concentration of each compound (**1**, **8**, **9,** and **11**) was 40 μM; Cisplatin (40 μM) and paclitaxel (5 μM) were used as positive controls. All experiments were conducted in triplicate, and data were expressed as the mean ± standard deviation of three independent experiments.

Compounds **1**, **8**, **9,** and **11** were assessed to determine their cytotoxic activity using the MTS method. During an initial active screening, compound concentration was used at 40 μM; the results showed that compounds **1**, **8**, **9,** and **11** had initial inhibitory rates of 21.5%, 100.0%, 15.4%, and 15.6%, respectively, against HL-60; 37.4%, 100.0%, 13.6%, and 0.33%, respectively, against A549 cells; 12.1, 100.0, 30.1, and 6.9%, respectively, against SMMC-7721; 37.9%, 95.5%, 24.4%, and 10.7%, respectively, against MDA-MB-231; and 41.2%, 100.0%, 21.6%, and 4.3%, respectively, against SW480 (Figure 4). Among them, the IC_50_ values of compound **8** were 12.89 ± 0.81 μM (HL-60), 15.69 ± 0.61 μM (A549), 3.00 ± 0.27 μM (SMMC-7721), 4.644 ± 0.270 μM (MDA-MB-231), and 14.02 ± 0.52 μM (SW480).

### 3.3 Inhibitory activity against protein kinase Cα

**Figure 5:**
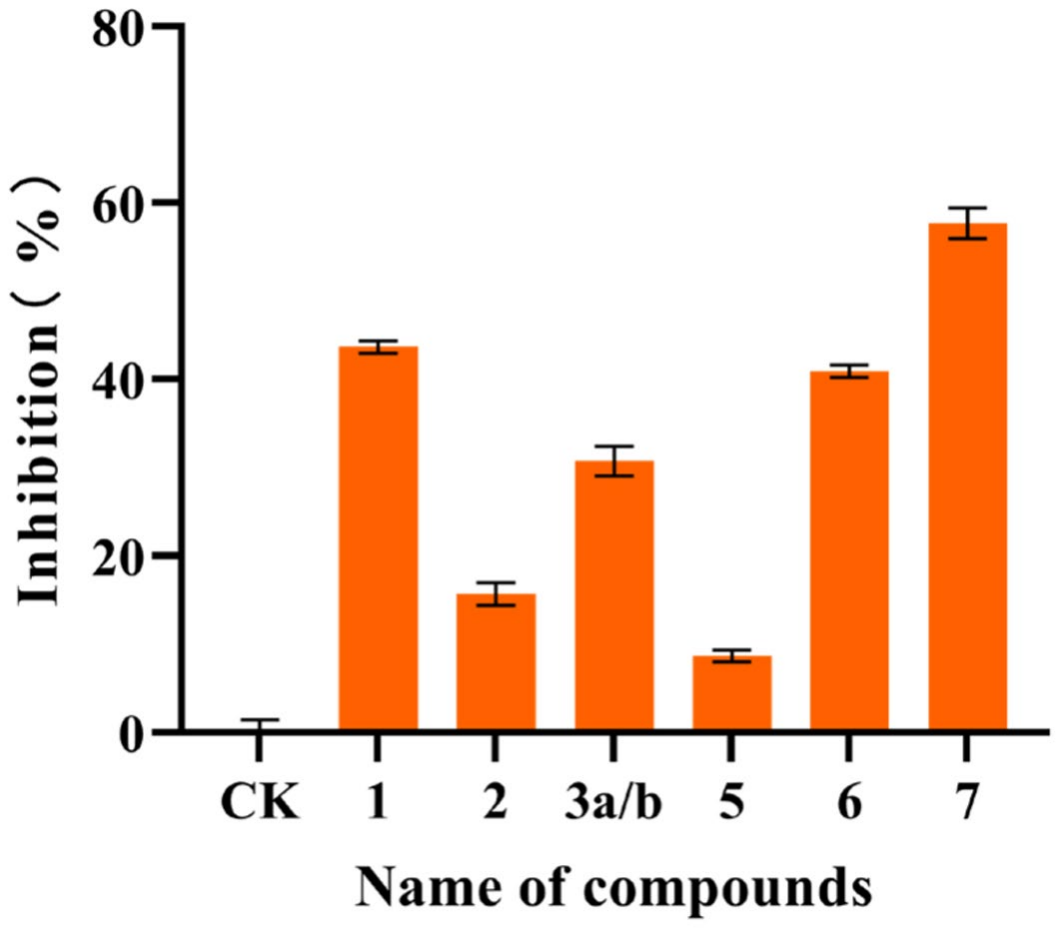
Inhibitory activity of some compounds on protein kinase Cα. The inhibitory activity of compounds against protein kinase Cα is determined based on the basic principles of ELISA. Compared with the control, the inhibition rates of the compounds **1**, **2**, **3a/b**, **5**, **6**, and **7** at a concentration of 20 μg/mL on protein kinase Cα were 43.63%, 15.68%, 30.77%, 8.68%, 40.93%, and 57.66%, respectively. All experiments were conducted in triplicate, and data were expressed as the mean ± standard deviation of three independent experiments.

In this study, the compounds were analyzed using the data obtained from the SwissTargetPrediction website, revealing that some of them had protein kinase Cα target sites. Subsequently, the effect of these compounds on protein kinase Cα inhibitory activity was assessed, which is determined based on the basic principles of ELISA. Compounds **1**, **2**, **3a/b**, **5, 6,** and **7** were evaluated at a concentration of 20 μg/mL. After substituting the OD values of the six compounds and the blank control, the actual activity concentrations of protein kinase Cα were determined. Compared with the control group, the protein kinase activity of the treated group was obtained. Then according to the inhibition rate formula, the inhibition rates of the six compounds on protein kinase Cα were 43.63%, 15.68%, 30.77%, 8.68%, 40.93%, and 57.66%, respectively. The activity results are shown in Figure 5. Results indicated that compounds **1**, **6,** and **7** had some inhibitory effect on protein kinase Cα activity.

## 4 Discussion

The investigation of stroma and host complexes of *Cordyceps* has consistently been a focal point in research. However, acquiring these complexes in substantial quantities remains challenging. Consequently, researchers have focused on exploring the activities and components of their anamorphs. In a previous study, we screened *P. gunnii* YMF1.00003 under different culture conditions through the conduct of metabolome analysis and activity comparison and found that the fermentation extract of the mycelium in WGA was similar to the extract of stroma and host complexes and had obvious cytotoxic activity (Qu et al., 2022). Therefore, we further examined the metabolites and activities of *P. gunnii* YMF1.00003 fermented in two different media. Eleven compounds (**1–11**) were identified, including **1**, **4**, **8,** and **11** from the modified WGA medium and other metabolites from the modified Sabouraud solid medium.

The extract of *P. gunnii* YMF1.00003 showed notable cytotoxic activity. Hence, several isolated compounds were evaluated to ascertain their cytotoxic activity. Compound **8** exhibited moderate cytotoxic activity against five tumor cell lines, with IC_50_ values of 3.00 ± 0.27 μM (SMMC-7721), 4.644 ± 0.270 μM (MDA-MB-231), 12.89 ± 0.81 μM (HL-60), 15.69 ± 0.61 μM (A549), and 14.02 ± 0.52 μM (SW480), respectively. Compound **1** showed weak cytotoxic activity against five tumor cell lines at a concentration of 40 μM. Ergosterols, particularly ergosterol peroxide, have been recognized for their potent cytotoxic activity (Zhabinskii et al., 2022; Hoang et al., 2023). According to the existing literature, the position of hydroxyl substitution, the position of double bonds, and the degree of oxidation are pivotal factors influencing cytotoxicity (Zhabinskii et al., 2022). The mechanism underlying the cytotoxic activity of this compound has been documented: ergosterol peroxide stimulates Foxo3 activity by inhibiting pAKT and c-Myc and activating the pro-apoptotic proteins Puma and Bax to induce cancer cell death (Li et al., 2016). Compound **1** is classified as a non-symmetric macrocyclic bislactone, and this type of bislactone has demonstrated certain antimicrobial bioactivity in previous studies (Krohn et al., 2007). SwissTargetPrediction was launched in 2014 and used as a web-based tool for predicting the potential protein targets of small molecules. It predicts the potential targets of all examined compounds based on their similarity with known bioactive compounds (Gfeller et al., 2014). However, virtual screening is frequently associated with high false-positive rates. Several compounds that receive high rankings for a specific target protein may not exhibit actual activity. During a typical virtual screening, only approximately 12% of the highest-scoring compounds demonstrated activity when subjected to biochemical analysis (Adeshina et al., 2020). Therefore, virtual screening serves as a crucial and beneficial tool, yet necessitating further experimental validation. In the present study, the STP was employed to identify targets associated with the metabolites (Gfeller et al., 2014). The results indicated a potential interaction between this type of bislactone and protein kinase Cα. Our active screening result showed that compounds **1**, **6,** and **7** inhibited the activity of protein kinase Cα by 43.63%, 40.93%, and 57.66% at a concentration of 20 μg/mL, respectively. Protein kinase Cα is implicated in various diseases, such as cardiovascular diseases (Turner et al., 2013), schizophrenia (Carroll et al., 2010), and the neural basis of episodic memory (MacLeod and Donaldson, 2014). However, due to the low inhibition rate of protein kinase Cα activity and the limited amount of compounds, further investigations were not pursued. Protein kinase C, a phospholipid- and calcium-dependent protein kinase, has been marketed as a drug target for multiple drugs, providing significant clinical benefits to patients with cardiovascular and cerebrovascular diseases, leukemia, and diabetes (Carter, 2000). The wild stroma and host complexes of *Cordyceps* fungus have been a focal point of research, but their scarcity has led researchers to concentrate on clonal mycelium. In the initial stages, we evaluated the *Paecilomyces gunnii* extracts under different culture conditions and from the wild stroma and host complexes through the performance of a metabolome analysis and identified the culture conditions for producing metabolites with enhanced cytotoxic activity (Qu et al., 2022). In the present study, fermentation was conducted under screened culture conditions to yield active natural products. The results showed that compound 3β-hydroxy-7α-methoxy-5α,6α-epoxy-8(14),22*E*-dien-ergosta (**8**) exhibited notable antitumor activity, while compounds **1**, **6,** and **7** displayed a certain inhibitory effect on protein kinase Cα.

With the rapid advancements in sequencing technology and bioinformatics, numerous fungal gene clusters have been found to remain “silent” under conventional culture conditions. This suggests the presence of a large number of potentially active compounds with novel structures that are concealed within these “silent” gene clusters and yet to be identified (Nielsen et al., 2017; Kjærbolling et al., 2019). The current study is based on a previous one stain-many compounds (OSMAC) strategy and the isolation and identification of promising compounds under optimized culture conditions (Qu et al., 2022). In recent years, the OSMAC strategy has proven successful in isolating compounds with novel structures and diverse activities from fungi (Bode et al., 2002). Following the screening of culture conditions, hydroxy-substituted fatty acids with nematicidal activity were obtained from *Purpureocillium lavendulum* (Liu et al., 2022). Diketopiperazine alkaloids were purified from a marine endophytic fungus *Penicillium* brocae with strong anti-*Staphylococcus aureus* activity and cytotoxic activity using the “OSMAC” strategy (Meng et al., 2016). The fungal genome harbors numerous transcription regulators, and the transcription and expression of functional genes are often controlled by these regulators. The discovery of new secondary metabolites can be facilitated by manipulating specific transcription factors (Keller, 2019). However, the efficacy of mining fungal metabolites through fermentation conditions remains contingent on factors such as whether the genomic data of the target fungus has been measured, the quality of the genomic data, and the establishment of genetic transformation.

## Data availability statement

The original contributions presented in the study are included in the article/supplementary material, further inquiries can be directed to the corresponding author.

## Ethics statement

Ethical approval was not required for the studies on humans in accordance with the local legislation and institutional requirements because only commercially available established cell lines were used.

## Author contributions

S-SL: Data curation, Investigation, Writing – original draft. S-LQ: Data curation, Investigation, Writing – original draft. JX: Investigation, Writing – original draft. DL: Investigation, Data curation, Writing – original draft. P-JZ: Data curation, Funding acquisition, Investigation, Resources, Writing – review & editing.

## Footnotes

^1^ http://www.swisstargetprediction.ch/
